# A Framework for Widespread Replication of a Highly Spatially Resolved Childhood Lead Exposure Risk Model

**DOI:** 10.1289/ehp.11540

**Published:** 2008-08-14

**Authors:** Dohyeong Kim, M. Alicia Overstreet Galeano, Andrew Hull, Marie Lynn Miranda

**Affiliations:** Nicholas School of the Environment, Duke University, Durham, North Carolina, USA

**Keywords:** children’s health, environmental justice, exposure risk prevention, geocoding, GIS (geographic information systems), lead

## Abstract

**Background:**

Preventive approaches to childhood lead poisoning are critical for addressing this longstanding environmental health concern. Moreover, increasing evidence of cognitive effects of blood lead levels < 10 μg/dL highlights the need for improved exposure prevention interventions.

**Objectives:**

Geographic information system–based childhood lead exposure risk models, especially if executed at highly resolved spatial scales, can help identify children most at risk of lead exposure, as well as prioritize and direct housing and health-protective intervention programs. However, developing highly resolved spatial data requires labor-and time-intensive geocoding and analytical processes. In this study we evaluated the benefit of increased effort spent geocoding in terms of improved performance of lead exposure risk models.

**Methods:**

We constructed three childhood lead exposure risk models based on established methods but using different levels of geocoded data from blood lead surveillance, county tax assessors, and the 2000 U.S. Census for 18 counties in North Carolina. We used the results to predict lead exposure risk levels mapped at the individual tax parcel unit.

**Results:**

The models performed well enough to identify high-risk areas for targeted intervention, even with a relatively low level of effort on geocoding.

**Conclusions:**

This study demonstrates the feasibility of widespread replication of highly spatially resolved childhood lead exposure risk models. The models guide resource-constrained local health and housing departments and community-based organizations on how best to expend their efforts in preventing and mitigating lead exposure risk in their communities.

Although much progress has been made, childhood lead poisoning remains a critical environmental health and justice concern. Lead causes irreversible, asymptomatic effects at levels far below those previously considered safe. The 2003–2004 National Health and Nutrition Examination Survey (NHANES) survey data reveal blood lead levels (BLLs) at or above the Centers for Disease Control and Prevention (CDC) blood lead action level of 10 μg/dL in 2.3% of 1- to 5-year-olds in the United States, with children tested having an overall geometric mean BLL of 2.1 μg/dL (National Center for Health Statistics 2006). These data indicate that > 500,000 children < 6 years of age experience elevated BLLs (EBLLs) at or above the CDC blood lead action level of 10 μg/dL (U.S. Census Bureau 2002).

Research suggests that significant adverse health effects occur at BLLs below the current CDC action level. Learning and behavioral deficits may occur even at BLLs < 5 μg/dL (Canfield et al. 2003; Chiodo et al. 2004; Lanphear et al. 2000; Miranda et al. 2007; Schnaas et al. 2006). Meta-analysis and reviews suggest that there is no threshold effect level, so any level of exposure is potentially detrimental (Gatsonis and Needleman 1992; Lanphear et al. 2005; Schwartz 1993, 1994). Thus, the number of children negatively affected by low-level lead exposure is likely much higher than estimates based upon the CDC action level. From a public health perspective, it is critical to get children who are at risk screened for lead in a timely manner and to intervene to rehabilitate the portions of the housing stock that pose the greatest risk of lead exposure.

Geographic information systems (GIS) hold particular promise for addressing the risks of childhood lead exposure. Wartenberg (1991) proposed a framework within which each type of screening approach—from health care provider and city clinic to door-to-door and finally to GIS-directed or informed screening—increases the case-finding rate. GIS has been used to evaluate known risk factors, such as old housing (Krieger et al. 2003; Reissman et al. 2001; Roberts et al. 2003), race (Haley and Talbot 2004), income (Krieger et al. 2003), and education (Haley and Talbot 2004; Krieger et al. 2003). In previous studies, addresses of children screened for lead and of children with EBLLs, as well as age of housing, have been used to determine screening rates and prevalence ratios of EBLLs (Reissman et al. 2001; Roberts et al. 2003). Knowing the spatial distribution of these rates allowed researchers to evaluate the effectiveness of screening programs and determine locations of greatest concern. Reissman et al. (2001) found that even in previously prioritized screening zones, only 50% of the children were being screened.

Although some models have been developed at more refined spatial scales (census tract or block group) (Krieger et al. 2003), most of the literature analyzes lead surveillance data at the ZIP code level. ZIP codes are useful because typically they are easily recognized by parents and health care providers, and with one simple question they can be used to identify children who should be tested for lead exposure. This simplified characterization, however, ignores the nonstandardized and highly dynamic structure of ZIP codes (Grubesic and Matisziw 2006), as well as spatial variability within ZIP codes (Matisziw et al. 2008). The key, then, is to pick an areal unit that is readily recognized by parents and health care providers and that is sufficiently disaggregated to maximize exposure risk insights.

In an effort to overcome the potential shortcomings of analyzing spatially aggregated data, some recent analyses of blood lead screening data have used highly refined geographic scales. Roberts et al. (2003) used household level data to identify an area of older houses where children with EBLLs live. Miranda et al. (2002) built models based on tax parcels to predict relative lead exposure risk levels for all residential parcels in a county, combining county tax assessor, North Carolina blood lead surveillance, and 1990 U.S. Census data.

Highly resolved models allow communities to target the highest-risk homes more cost-effectively and to create and implement targeted intervention programs. For example, using the lead exposure risk model based on tax parcels described by Miranda et al. (2002) led to a 600% increase in the capture rate of children with EBLLs in Durham County, North Carolina. Local health departments use the models to cross-reference high-risk housing with Medicaid and WIC (Women, Infants, and Children) recipients, enabling them to comply with state lead testing mandates and thus protect these especially vulnerable children. Local health departments also cross-reference recent births to determine whether infants are residing in high-risk housing and would thus benefit from early lead education interventions. Local housing departments use the model to recruit homeowners into lead abatement and rehabilitation programs. The models are also used by community groups in planning activities, such as door-to-door outreach, health fairs, and blood lead screening events.

At the state level, these models are used by the North Carolina Lead Poisoning Prevention Program (2008) to recruit property owners into the North Carolina Preventive Maintenance Program (North Carolina Childhood Lead Poisoning Prevention Program 2008) and to enforce disclosure provisions of the federal Title X, Residential Lead-Based Paint Hazard Reduction Act of 1992. In a clinical setting, health care providers can use the models to understand the risk of exposure to lead-based paint hazards in the individual patient’s home and day care center. With this information, the provider can decide whether to test a child for lead exposure and, perhaps more important, educate the parents regarding exposure prevention strategies. The maps that result from the models can serve as very effective tools to capture the interest of patients by personalizing the message about risk and prevention.

Of course, these highly resolved spatial models require additional expenditure of time and effort spent geocoding to the tax parcel level. Here, we explicitly examine how much effort needs to be expended on geocoding blood lead surveillance data to build a well-functioning and effective tax parcel–level model. In so doing, we demonstrate a framework for the widespread replication of this highly spatially resolved childhood lead exposure risk model across North Carolina and nationally. Because it is equally interpretable from a housing and health perspective, the more highly resolved spatial model provides a basis for improving the efficiency of blood lead surveillance programs and, perhaps more important, for shifting lead exposure programs from a mitigative to a preventive paradigm by directly influencing health education and housing intervention programs.

## Materials and Methods

**Figure 1:**
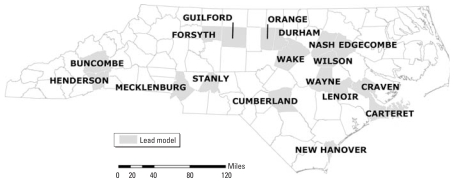
Map of 18 counties in North Carolina included in the analysis.

We updated Miranda et al.’s (2002) analysis using additional blood lead surveillance and updated tax parcel data from 18 North Carolina counties and by substituting 2000 Census data for the 1990 data used in the earlier analysis (U.S. Census Bureau 2000, 2002). The 18 counties span the state and represent very different populations, climates, economies, and housing stocks. They include Buncombe, Carteret, Craven, Cumberland, Durham, Edgecombe, Forsyth, Guilford, Henderson, Lenoir, Mecklenburg, Nash, New Hanover, Orange, Stanly, Wake, Wayne, and Wilson Counties, as shown in Figure 1. Methods for receiving, storing, linking, and analyzing data and presenting results related to this study were all governed by a research protocol approved by the Duke University Institutional Review Board.

The models include demographic data from the 2000 Census at the block group and block level (U.S. Census Bureau 2000). We overlaid the Census data on publicly available tax assessor data from each of the counties. In the model, we focused on residential tax parcels, which typically include single-or multifamily housing structures. Digital tax assessor data vary from county to county, but models for each county include year of construction.

We characterized the relationship between BLLs and housing and demographic characteristics by geocoding to the tax parcel the blood lead test results for children who were 9 months to 6 years of age and who were tested between 1995 and 2003. Eighty-nine percent of blood lead samples were capillary draws, with only 5% reported as venous draws. The sample collection method was reported as unknown for the remaining 6% of lead test results. Access to the blood lead data was granted via a negotiated confidentiality agreement with the North Carolina Childhood Lead Poisoning Prevention Program. We geocoded the lead surveillance data to the individual tax parcel unit (as opposed to larger geographic units such as census block groups or tracts) using the tax assessor databases.

Parcel geocoding is a critical step in developing these highly resolved spatial models. Geocoding refers to the process of assigning a geographic coordinate (latitude and longitude) to observations from one data set (in this case, blood lead screens) using reference data (in this case, tax parcel data). This process facilitates the linking of multiple data sets via spatial relationships. Thus, we linked an environmental exposure biomarker (BLL test results) to a polygonal areal unit (tax parcel) via the residential address common to both data sets.

We had 467,204 BLL test results for this population, representing 336,736 individual children. We attempted to geocode all records with complete addresses, defined as addresses with at least street number, street name, and street type, to county tax parcel data. Parcel address information varied by county in both quality and level of completeness. Quality of surveillance address information was also not uniform across local health departments, clinics, and laboratories. Percentages of records geocoded by county ranged from 42.5% to 89.0% for all records and from 56.1% to 96.2% for records with complete addresses. Tax assessor address data tend to be of poorer quality for housing authority parcels and other multifamily complexes, leading to lower match rates for children who reside in these tax parcels.

We implemented three stages of geocoding, as described in Table 1. Level I geocoding was an exact match of “as-reported” address information to reference parcel data. With the North Carolina lead screening data, we geocoded about 36.4% of all records by the level I process, which took 7–9 days with one trained staff member working 8 hr/day. Level II geocoding matched data after standardizing the lead screening data to reflect the reference data structure (e.g., by converting all versions of “street”—str., street, st, etc.—to ST). This process took about 20 days and yielded an additional 10.4% of records. Level III geocoding processed records, one by one, using visual analysis of and matching to tax parcel address data. This stage required an additional 3–4 months and led to an additional 22.0% of records being geocoded. Even after the most intensive level III geocoding, 31.2% of records remained ungeocoded, although one-third of these did not include a complete address.

In general, the level I stage geocoded records rapidly, but in some counties the number geocoded by this process alone may not be sufficient. Level II geocoding provided additional data with little additional effort, whereas level III geocoding required substantial time and effort and might be more prone to errors in positional accuracy—that is, locating an observation at the wrong parcel. This could be due to the quality of the recorded address or to the process. Geocoding outcomes differ substantially across individual counties. For instance, the level III process geocoded approximately 40% of records in Carteret and Lenoir Counties but no records in Henderson County.

After all three geocoding processes were complete, we included only the record with the highest BLL at each parcel for each child in the analysis. We used this conservative or more protective selection method because the highest results provide information regarding levels of exposure to biologically available lead. This method is consistent with the approach used by Lanphear et al. (1998). Because of left skewness, the natural logarithm of BLLs from the blood lead data served as the dependent variable in our multivariate statistical analysis, in which we used a weighted regression model to avoid having model output influenced excessively by tax parcels with multiple records. We performed the regression with clustering by block group to adjust standard errors for correlation within the same block group. Explanatory variables included median household income, percentage of households receiving public assistance, percent African Americans, and percent Hispanics—all taken from the 2000 U.S. Census. We also used year of construction from the tax assessor data and accounted for seasonal changes in lead exposure by including three dummy variables for seasons when the blood samples were taken (winter as reference) (Miranda et al. 2007). We also included dummy variables for each of the counties. We combined the spatially linked data listed above into a single GIS database to prepare for statistical analysis using ArcGIS, version 9.1 (ESRI, Redlands, CA).We performed statistical analysis using Stata, version 9.0 (StataCorp., College Station, TX).

We ran three models using combinations of data from the different geocoding processes: *a*) level I geocoding only, *b*) levels I and II geocoding, and *c*) levels I–III geocoding. We compared the results from the models to investigate how much the additional data from more intensive geocoding processes improve performance of childhood lead exposure risk models in identifying areas of elevated lead exposure risk.

## Results

The results of the revised model in the additional counties are consistent with findings reported previously (Miranda et al. 2002). Table 2 compares the results of the original six-county model with the three versions of our 18-county model. The marked decrease in the coefficient of median income in the 18-county models compared with the original six-county model could result from adding more county-specific dummies where some income effects are embedded. More likely, the change may be due to income growth in the North Carolina counties between the 1990 Census and the 2000 Census, in which income is expressed in nominal rather than real terms. A Wilcoxon matched-pairs signed-ranks test confirmed that the two income distribution functions are identical but differ only with respect to their median values. The coefficients for age of housing and percent African American are relatively close between the 6-county and 18-county models. The new variables—percent Hispanics, percentage of households receiving public assistance, and dummies for seasonality—were all significant and of the expected sign.

Table 2 also shows that the 18-county models provide very similar results across the three levels of geocoding. Although using a substantially smaller number of observations, the models using only level I geocoding and level I and II geocoding produced very similar coefficients and model fit compared with the model using levels I–III geocoding, except for a minimal difference for some county-specific dummies. This finding is noteworthy because it implies that additional time and resources dedicated to level III geocoding do not significantly alter or affect statistical modeling results.

We then applied the coefficient vector to the corresponding variables at the tax parcel level, creating a lead exposure risk index value for each residential tax parcel unit in each of the 18 counties, as the data permitted. (Although we conducted the model analysis using only those tax parcels that had been linked to a blood lead screen, the resulting model parameters can be applied to all residential tax parcel units.) We coded all tax parcel units in each county that could be identified as residential into one of four priority categories based on the relative size of their risk index values: top 10%, 10–20%, 20–60%, and 60–100%. We chose this “10–10–40–40” display structure based on several conversations with local health and housing department personnel regarding how to display model outputs in ways that are meaningful and directly useful to policy makers and other stakeholders.

**Figure 2:**
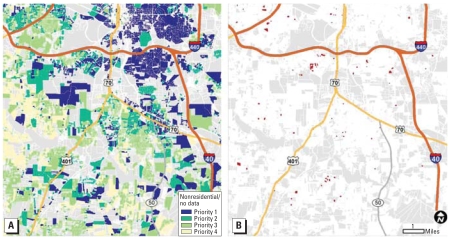
Lead risk priority maps and model performance for a portion of Wake County, North Carolina. (*A*) Map with level I + II data: priority 1 parcels predicted most likely to contain lead-based paint hazards (top 10%); priority 2 and 3 parcels (10–20% and 20–60%); priority 4 parcels (60–100%), least likely to contain lead-based paint hazards. The white areas are nonresidential parcels or parcels for which we have no data (missing year of construction or suppression of data by the U.S. Census for confidentiality reasons). (*B*) Map showing only the parcels where priorities change with level III data (red).

Figure 2A maps the 10–10–40–40 exposure risk priorities for a portion of Wake County, North Carolina, based on the results from the models using data from level I and II geocoding. This is only one example of how model results can be grouped and displayed. The models allow for flexibility and adaptation to different intervention strategies. This model does not capture non-housing-related lead exposure risk, such as cosmetics, toys, or traditional medicines.

For all of the counties included in this analysis, the ranks of risk index created by the three models are highly correlated, and the Spearman correlation coefficient is close to 1. Most parcels remain unchanged in their risk priority with additional data from more intensive geocoding; the risk categories change only for < 1% of the parcels. In Wake County, for instance, the two models assign different categories to only 1,095 of the total 198,045 parcels (Figure 2B).

**Figure 3:**
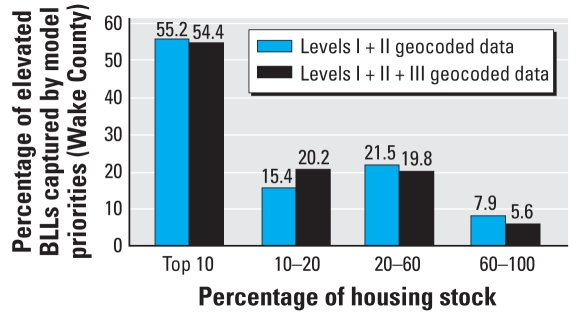
EBLLs within housing stock priority categories.

Figure 3 summarizes the distribution of EBLLs (≥10 μg/dL) by priority risk categories from the models with versus without level III data for Wake County. An intervention program based on the model with level I and II data that targeted only the top 10% of the housing stock in Wake County would have captured 55.2% of the EBLLs in the county, with an additional 15.4% of EBLLs captured by targeting the next 10% of the housing stock. This compares very favorably with the 54.4% and 20.2% that would have been captured by targeting the top 10% and next 10%, respectively, of the housing stock using the full model with data from levels I, II, and III. We found similar results for all 18 counties in this analysis, indicating that the lead exposure risk model based on level I and II geocoding performs on par with the model based on levels I–III geocoding, with considerably less effort expended on developing the model. This finding supports the feasibility of widespread replication of a highly spatially resolved childhood lead exposure risk model.

## Discussion

Comparing the model using level I and II data with that using levels I–III data shows that, for most counties, expending the additional effort on level III geocoding adds little to no improvement in model performance. The overall performance of these models tends to be driven at least partly by the underlying quality of address data in both the tax parcel and the lead surveillance data sets, as well as the total number of children previously screened in the county. Tax parcel data-quality improvements are rapid as local governments realize the gains in efficiency in property tax collection made possible by high-quality digitized parcel data. Lead surveillance data are stored in a central registry in North Carolina (North Carolina Childhood Lead Poisoning Prevention Program, North Carolina Department of Environment and Natural Resources databases, unpublished data), which is extremely helpful for developing models for multiple counties at the same time. In addition, the quality of these data has improved substantially, as have the postcollection quality assurance and quality control practices at state offices. The number of children who have previously been screened—and whose data thus serve as the basis for building the childhood lead exposure risk models—varies from county to county, with much lower levels in more sparsely populated counties, as would be expected. In these situations, additional years of data may need to be included to improve the performance of the models.

We have replicated the parcel-level lead exposure risk models for Kenosha County, Wisconsin, and for the City of Detroit, Michigan (Miranda ML, Kim D, Galeano MAO, unpublished data) and found that the key explanatory variables in the North Carolina models remain significant in these two additional locales. The Kenosha model, on average, performs better than the North Carolina model, addressing 67% of the elevated cases by targeting the top 10% of the housing stock and 82% by targeting the top 20%. On the other hand, the performance of the Detroit model was not as good as the North Carolina or the Kenosha models, addressing 25% by targeting the top 10% and 46% by targeting the top 20%. This indicates that the sources of childhood lead poisoning may be more complex in heavily industrialized areas such as Detroit. Thus, the Detroit model includes variables to account for proximity to industrial facilities that either presently or historically emit lead. In addition, we are in the process of obtaining additional data layers related to industrial facilities that should substantially improve model performance.

The limitations of this study deserve mention and consideration for future research. First, the screens that remained ungeocoded even after level III efforts might have had an impact on the model results. Second, errors and inaccuracies associated with our three levels of geocoding, such as address matching errors, could have potentially affected the model parameters (Rushton et al. 2006). Third, although our study provides a framework for producing highly spatially resolved models for childhood lead exposure risk, in some geographic areas additional explanatory variables may be needed, as was the case, for example, in Detroit.

Despite these limitations, the potential for community outreach is great with these highly spatially resolved models. When enhanced by data layers of community resources, the model can be used to identify venues for outreach activities such as child care centers, places of worship, and community centers—particularly those located in high-risk areas. In one application of the model in Durham County, North Carolina, we are overlaying the childhood lead exposure risk model with a map of recent births. The health department can then use these maps and databases to direct a home visitation program that provides education on lead-safe cleaning practices, conducts home testing, and reminds parents to have their children screened for lead in a timely fashion. This approach gets critical information to families before children enter the developmental stage with high hand-to-mouth activity that exacerbates exposure to lead in the home.

We are also working to make the results of these models universally accessible through a Web-based portal. With this tool, a health service provider (or any community member) can look up an address to evaluate the associated lead risk at the residence, as well as other relevant public and environmental health concerns. This approach to GIS mapping allows for detailed evaluation of risks, while simultaneously protecting patient confidentiality. This Web-based approach helps identify children at risk quickly and makes targeted intervention more effective. In addition, it serves as a critical planning tool for housing departments and affordable housing coalitions, as they consider priorities for rehabilitating the housing stock. It has also been used to direct housing inspections to more quickly identify substandard houses. In a clinic setting, the model maps of those areas where patients live can be displayed in poster format to give patients (and health care providers) a sense of the exposure patterns for different residential areas. In addition, the Web-based direct look-up function can be integrated with health record databases to provide an automatic flag to health care providers to screen children for lead. Eventually, the portal could expand to include prevention of multiple potential residential exposures.

## Conclusions

This analysis demonstrates that widespread replication of highly spatially resolved childhood lead exposure risk models is feasible in any area with digitized tax parcel data. Increasingly, such data sets are being maintained digitally, even in low-income areas, as the cost of electronic data storage decreases and technical expertise increases. For example, in North Carolina, 84 of 100 counties already have digitized parcel data, with plans moving forward rapidly in the remaining 16 counties. Recent developments in geocoding techniques and reference data sets (e.g., parcel geocoding and building-level geocoding) will make highly spatially resolved data increasingly available to researchers and policy practitioners interested in using these technologies to improve public health.

## Figures and Tables

**Table 1:** Geocoding processes for 18 North Carolina counties.

Geocoding level	Description	No. of screens in 18 counties (1995–2003)	Time invested
I	Exact match of “as reported” address to reference (parcel) data	170,277 (36.4% of all records)	7–9 days
II	Exact match after address standardization to reflect reference data structure	48,459 (10.4% of all records)	20–22 days
III	Match using visual analysis of tax parcel data	102,854 (22.0% of all records)	90–120 days
Ungeocoded	Remain ungeocoded after level III geocoding	145,614 (31.2% of all records)	—

**Table 2:** Results of statistical models [dependent variable: ln(BLL) μg/dL].

		18-County model		
Independent variable	6-County model^a^ (*n* = 11,523)	Level I geocoding only (*n* = 122,674)	Level I + II geocoding (*n* = 156,461)	Level I + II + III geocoding (*n* = 214,878)
Year built	−0.0044	−0.0038	−0.0038	−0.0038
Household median income	−4.42 × 10^−6^	−1.83 ×10^−6^	−1.77 ×10^−6^	−1.75 ×10^−6^
Percent African American	0.002	0.0027	0.0027	0.0027
Percent Hispanic		0.0023	0.0024	0.0023
Percent public assistance		0.0040	0.0036	0.0034
Screened in spring		0.02	0.03	0.03
Screened in summer		0.08	0.09	0.09
Screened in fall		0.07	0.07	0.07
Buncombe County	9.85	8.58	8.43	8.51
Carteret County		8.67	8.52	8.60
Craven County		8.66	8.52	8.60
Cumberland County		8.64	8.49	8.57
Durham County	9.81	8.47	8.32	8.39
Edgecombe County	10.10	8.79	8.65	8.72
Forsyth County		8.63	8.48	8.55
Guilford County		8.59	8.45	8.52
Henderson County		8.68	8.55	8.62
Lenoir County		8.75	8.61	8.68
Mecklenburg County		8.59	8.44	8.51
Nash County		8.74	8.58	8.65
New Hanover County	9.92	8.63	8.48	8.55
Orange County	9.93	8.60	8.45	8.52
Stanly County		8.81	8.66	8.73
Wake County		8.60	8.45	8.52
Wayne County		8.71	8.56	8.63
Wilson County	10.30	8.73	8.58	8.67
Root mean square error	0.619	0.601	0.601	0.602

All coefficients are significant at the 1% level.

a Data from Miranda et al. (2002).
